# Optimized guidelines for feminized seed production in high-THC *Cannabis* cultivars

**DOI:** 10.3389/fpls.2024.1384286

**Published:** 2024-10-30

**Authors:** Antonio A. Timoteo Junior, Iain W. H. Oswald

**Affiliations:** ^1^ Department of Agriculture, Food, and Resource Sciences, University of Maryland Eastern Shore, Princess Anne, MD, United States; ^2^ Department of Research and Development, Abstrax Tech, Tustin, CA, United States

**Keywords:** Cannabis sativa, high-THC cultivars, delta-9 tetrahydrocannabinol, dioecious, plant sex reversal, seed feminization

## Abstract

With the partial legalization of high-THC *Cannabis sativa* across 23 states for recreational use and 38 states for medical purposes in the United States, the *Cannabis* industry is poised for significant growth. Projected to reach a sales volume of $50.7 billion by 2028, this growth is driven by the trend of lifting *Cannabis* prohibition. High-THC *C. sativa* cultivars, containing more than 0.3% delta-9 tetrahydrocannabinol (Δ9-THC) as defined by the 2018 US Farm Bill, are used for both medicinal and recreational purposes. *Cannabis sativa* is a short day, dioecious, annual plant, where female plants are favored for THC production, which requires seed feminization techniques to ensure an accurate female plant population. This involves using an ethylene inhibitor to induce sex reversal, leading to male flower development on female plants, allowing for self-pollination and the production of feminized seeds. However, challenges such as seed viability and the occurrence of male flowers in progeny have been noted. This review provides guidelines to enhance the production of viable feminized seeds in high-THC *Cannabis* cultivars. Literature findings indicate that Silver Thiosulfate (STS) is the most effective ethylene inhibitor for sex reversal and seed feminization in high-THC *Cannabis* cultivars. Specifically, a single dose of 3 mM STS should be applied during the vegetative stage via foliar spraying until runoff, followed by exposure to a short photoperiod of up to 12 hours to induce flowering and seed production. Progeny plants should be assessed for seed germination rate and compared for growth performance with the original parent plant to assess the declining effects of inbreeding. Adhering to these guidelines can improve the quality and viability of feminized seeds, meeting commercial market standards and industry demands for high-THC *Cannabis* cultivars.

## 1 Introduction

High-THC *Cannabis*, commonly referred to as marijuana, denotes any *Cannabis sativa* L. cultivar containing more than 0.3% delta-9 tetrahydrocannabinol (Δ9-THC), the compound responsible for its psychoactive effects as defined by the 2014 and 2018 US Farm Bills (House of Representatives, C, 2018). In contrast, cultivars with up to 0.3% Δ9-THC are classified as industrial hemp, distinguishing drug-type (marijuana) from fiber-type (hemp) in accordance with the same regulations. Despite this distinction, both hemp and marijuana belong to the Cannabaceae family and share characteristics such as being annual, short-day, and dioecious plants (Junior et al., 2023). Dioecious refers to plants in which male and female gametes are produced by separate individual plants (Chandra et al., 2017; Monthony et al., 2021). While hemp enjoys complete legalization in the United States and serves various purposes, including food, feed, fiber, and non-psychoactive cannabinoid production, marijuana is legalized for medicinal and recreational use in select states (Chandra et al., 2017; Monthony et al., 2021; NCSL, 2023; Junior et al., 2023). Overall, Cannabis sales are projected to reach volume of $50.7 billion by 2028, this growth is driven by the trend of lifting Cannabis prohibition (Dorbian, 2023).

The therapeutic benefits of *Cannabis* are attributed to its diverse array of phytochemicals, including cannabinoids, terpenoids, non-terpenoids and others with various chemical functionalities (Oswald et al., 2021; Oswald et al., 2023). These compounds have shown promise in treating conditions such as epilepsy, breast cancer, asthma, and Alzheimer’s disease, as well as possessing antifungal, analgesic, antibacterial, and anti-inflammatory properties (Gertsch et al., 2008; McAllister et al., 2012; Vuolo et al., 2015; Gulluni et al., 2018; Cox-Georgian et al., 2019; Koo et al., 2020; Robaina-Cabrera et al., 2021).

Phytochemicals are primarily concentrated in trichomes, extracellular structures found on female flowers, making female plants more suitable for phytochemical production (Lubell and Brand, 2018; Sommano, 2020). Female *Cannabis* plants can be propagated sexually via seeds or asexually through methods like soft-wood cuttings and tissue culture (Chandra et al., 2017; Monthony et al., 2021). While sexual propagation is more economical for large-scale commercial production, regular seeds pose limitations as they produce both male and female plants (Lubell and Brand, 2018; DiMatteo et al., 2020; Yoshimatsu et al., 2010). Failure to remove male plants before pollen release can lead to cross-pollination and diminished phytochemical yields (Small, 2015).

To maximize THC content and exclude male plants, commercial *Cannabis* facilities often utilize feminized seeds (Small, 2015; Lubell and Brand, 2018; Kurtz et al., 2020, 2020b). However, challenges persist, as commercially certified feminized seeds may not always yield satisfactory results. For instance, a grower in Montana state encountered substantial economic losses when approximately 30% of the plants obtained from certified feminized seeds turned out to be male (Olejar and Park, 2022). This unintended occurrence led to cross-pollination and subsequent seed production, resulting in decreased THC levels causing economic losses (Olejar and Park, 2022).

This review aims to address these challenges and presents crucial seed feminization guidelines based on seven principles such as (I) the right stock (mother) plant material, (II) the right ethylene inhibitor to enhance sex reversal, (III) the right dosage of ethylene inhibitor, (IV) the right placement of the compound within the plant, (V) the right application frequency of the compound, (VI) the right photoperiod to induce flowering, and (VII) the right management of progeny plants, aiming to enhance feminized seed production in high-THC cultivars to meet commercial standards for both medical and recreational purposes.

## 2 Seed feminization in *Cannabis sativa* L

Feminized seeds produce exclusively female plants with all-female flowers, achieved by applying an ethylene inhibitor to female plants (Lubell and Brand, 2018). This induces sex reversal, prompting the development of male flowers on the female plants (Kurtz et al., 2020b; Flajšman et al., 2021). Ethylene, a naturally occurring ripening phytohormone, typically promotes the formation of female flowers (Lubell and Brand, 2018). However, when female *Cannabis* plants are treated with ethylene inhibitors such as aminoethoxyvinylglycine (AVG), cobalt nitrate (CBN), colloidal silver, silver thiosulfate, 1-Methylcyclopropene (1-MCP), they undergo sex reversal, becoming monoecious or bisexual (Lubell and Brand, 2018; Flajšman et al., 2021; Fitzgerald, 2021). In this state, they bear both male and female flowers on the same plant, facilitating self-fertilization and seed production (Lubell and Brand, 2018; Flajšman et al., 2021). The resulting seeds exclusively carry the XX female chromosome inherited from the original self-crossed female parent, ensuring that all plants derived from these seeds are exclusively female, capable of producing all-female flowers plant (Lubell and Brand, 2018; DiMatteo et al., 2020; Kurtz et al., 2020b; Flajšman et al., 2021).

## 3 The role of phytohormones in plant sex reversal

Plant growth hormones such as ethylene, auxins, and cytokinin promote the development of female flowers in *Cannabis*, while gibberellins stimulate the formation of male flowers; controlling these phytohormones can partially reverse plant sex (Galoch, 1978; Yamasaki et al., 2005; Lubell and Brand, 2018). For instance, Flajšman et al. (2021) investigated the impact of foliar application of silver thiosulfate and colloidal silver on sex reversal and masculinization of female *Cannabis* plants, resulting in the induction of male flowers, self-pollination of the remaining female flowers, and production of feminized seeds. Similarly, Lubell and Brand (2018) observed that silver thiosulfate foliar application induced male flowers on treated female plants under short photoperiod conditions. Additionally, apical application of aminoethoxyvinylglycine (AVG) promoted the development of male flowers in lateral branches of dioecious female plants (Mohan-Ram and Sett, 1982).

Sex reversal in *Cannabis* plants is not exclusive to female plants; male plants can also undergo sex reversal by inducing female flowers for seed production (Moon et al., 2020). However, the offspring will exhibit male plants due to the inheritance of the parental heterogametic Y chromosome (Moon et al., 2020). For instance, male plants treated with ethephon foliar spray developed female flowers (Moon et al., 2020).

## 4 Sex determination in *Cannabis*

Manipulating phytohormones to induce plant sex reversal does not alter the underlying genetic framework governing sex determination in *Cannabis* plants. In *Cannabis*, sex determination is primarily regulated by genetic factors rather than phytohormonal influence (Yamasaki et al., 2005; Lubell and Brand, 2018). Two main mechanisms dictate sex determination in *Cannabis*: the active Y-chromosome pathway and the X-to-autosome (X: A) balance system (Vyskot and Hobza, 2004; Yamasaki et al., 2005). In the active Y-chromosome pathway, specific genes carried by the Y chromosome suppress female sex expression, leading to stamen development and anther maturation (Yamasaki et al., 2005). Conversely, the X: A balance system regulates sex determination based on the ratio of X chromosomes to autosomes (Vyskot and Hobza, 2004; Yamasaki et al., 2005). A ratio equal to or higher than one promotes female sex expression, while a lower ratio (up to 0.5) favors male sex determination (Yamasaki et al., 2005). Additionally, intersex and hermaphroditism occur when the X:A ratio falls between 0.5 and 1.0 (Negrutiu et al., 2001; Vyskot and Hobza, 2004; Yamasaki et al., 2005). Furthermore, advancements in genomic approaches have facilitated the identification or prediction of individuals’ sex expression in *Cannabis* plants. Notably, the *Cannabis* Sex Purity-1 (CSP-1) assay has emerged as a promising tool for accurately predicting plant sex, particularly in its dioecious populations (Galoch, 1978; Laverty et al., 2019; Toth et al., 2020);. Its efficacy extends across various *Cannabis* varieties, including CBD (Cannabidiol) types, grain types, and grain/fiber types (Toth et al., 2020; Laverty et al., 2019). However, uncertainties persist regarding its accuracy, as evidenced by reported recombinants in previous assays (Divashuk et al., 2014; Törjék et al., 2002).

Recent insights from *Cannabis* whole-genome sequencing have highlighted potential genetic markers, such as *MADC6*, which may contribute to the success of the *CSP-1* assay (Laverty et al., 2019). The discovery of unassembled scaffolds in the male genome bearing high similarity to the *MADC6* sequence underscores the potential of genomic approaches to enhance sex determination accuracy in *Cannabis* plants (Sakamoto et al., 2000; Vyskot and Hobza, 2004). Moreover, this approach emphasizes the intricate nature of sex determination in *Cannabis*, involving genetic factors like the active Y-chromosome pathway and the X-to-autosome balance system (Sakamoto et al., 2000; Vyskot and Hobza, 2004). While molecular assays like the *CSP-1* sex assay provide valuable insights, a comprehensive understanding of the genetic mechanisms underlying sex determination is essential.

## 5 Crucial principles for enhanced seed feminization in high-THC cultivars

### 5.1 The right stock (mother) plant

Choosing the ideal mother plant for feminized seed production in *Cannabis* is crucial for achieving high-quality yields. Breeders prioritize traits such as plant health, desired characteristics (height, biomass), and optimal THC levels to pass on to the next generation (Zhang et al., 2020; Andre et al., 2016).

As an example of choosing the correct stock, a cultivator experienced significant losses when approximately 30% of plants grown from certified feminized seeds turned out to be male, resulting in cross-pollination and negative impacts on neighboring growers (Olejar and Park, 2022). This scenario can arise from two potential causes: either the mother plants used for seed feminization production were not all female plants, or male pollen drift occurred from the neighborhood. It is important to note that feminized seeds or feminized progeny plants cannot carry the Y chromosome unless it was present in the genetic makeup of the original parent plant (Galoch, 1978; Lubell and Brand, 2018).

**Figure 1:**
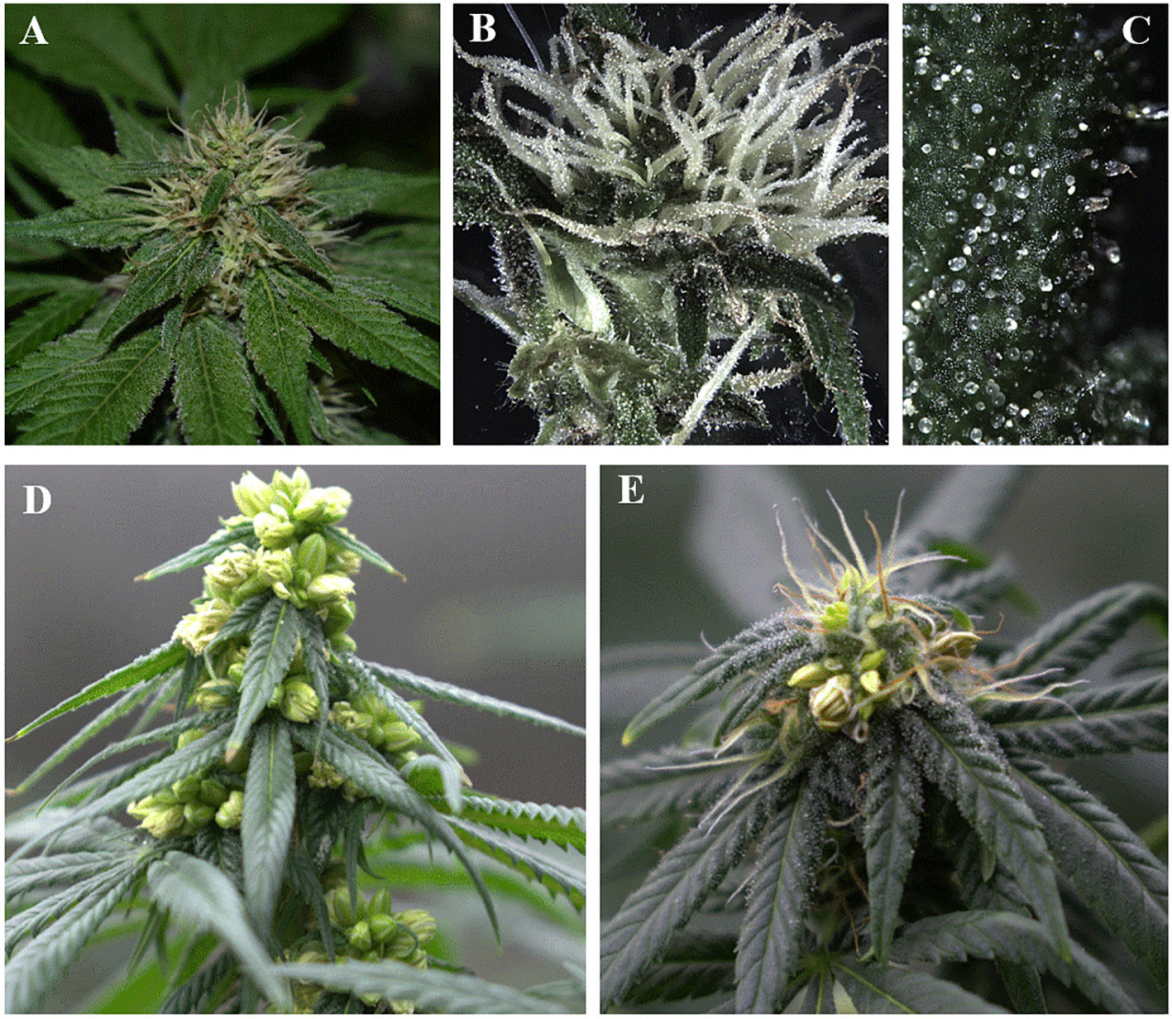
Overview of *Cannabis sativa* inflorescence: **(A)** Female flower prior to bud formation; **(B)** Stigma of female flowers; **(C)** Trichomes, mostly abundant in female inflorescence where phytochemicals are accumulated; **(D)** Male flowers during dehiscence; **(E)** Monoecious inflorescence expressing both male and female flowers in the same individual plant, which will then self-pollinate, fertilize, and produce feminized seeds that express all-female flowers.

To avoid this issue, it is imperative to ensure the female sex of the mother plant by inducing it to flower and ensuring that each node exclusively bears female flowers, without any male ones (**Figure 1**). Additionally, sex identification of mother plants can be confirmed through molecular methods like qPCR (Torres et al., 2022).

To prevent pollen drift, the feminization process should be conducted indoors to prevent cross-pollination with neighboring male plants. The mother plant must be strictly genetically female, expressing only female flowers, to ensure the presence of the XX chromosome in subsequent generations.

### 5.2 The right ethylene inhibitor for sex reversal

There are several studies regarding the most efficient chemical compound for enhancing sex reversal in *Cannabis*. For instance, in a study by Flajšman et al. (2021), the effects of silver thiosulfate (STS), gibberellic acid (GA), and colloidal silver on the masculinization of female plants in hemp cultivars were examined. The results indicated that both STS and colloidal silver compounds were effective in inducing the development of male flowers on female plants, while GA was ineffective. Similarly, another study by Rafiq et al. (2021) compared STS and GA to masculinize female *Cannabis* plants. Their findings showed that female plants treated with STS induced more male flowers and produced more viable pollen compared to those treated with GA. Additionally, Mohan-Ram and Sett (1982) demonstrated that STS was more effective than silver nitrate (AgNO3) in inducing male flowers of altered sex *Cannabis* plants. Moreover, Fitzgerald (2021) reported that *Cannabis* female plants treated with STS exhibited superior masculinization and pollen dispersal compared to plants treated with aminoethoxyvinylglycine (AVG), cobalt nitrate (CBN), and 1-methylcyclopropene (1-MCP). Also, the application of silver thiosulfate to genetically female plants of four hemp cultivars resulted in the production of male flowers (Lubell and Brand, 2018).

Unarguably, these findings suggest that silver thiosulfate (STS) compound is the most consistent ethylene inhibitor for inducing maleness in female hemp plants and can be adopted for high THC marijuana cultivars. A silver thiosulfate (STS) solution can be prepared by mixing silver nitrate (AgNO3) and sodium thiosulfate (Na2S2O3) stock solution to a molar ratio of 1:4 (Cameron and Reid, 1981).

### 5.3 The right doses of silver thiosulfate

The degree of masculinization of female plants is directly proportional to the concentration of silver thiosulfate (STS) applied to the plants; the higher the STS concentration, the greater the number of male flowers and the fewer female flowers per individual plant (Lubell and Brand, 2018; Flajšman et al., 2021). For instance, a study where *Cannabis* plants were treated with 0.3 mM and 3 mM of STS showed that 3.0 mM STS produced more male flowers compared to 0.3 mM (Lubell and Brand, 2018; DiMatteo et al., 2020; Kurtz et al., 2020b). However, another study where *Cannabis* plants were treated with 0.7 mM and 20 mM STS did not show a significant difference in the number of male flowers per plant (Flajšman et al., 2021), although there was a slight increase. Since male flowers contain pollen grains and female flowers (pistil) contain stigma (a pair of white hairs) and ovary (prospective seed), stigma works as a pollen catcher, while fertilization and seed production take place in the ovule. Therefore, having fewer female flowers per plant reduces seed production per individual plant (UNODC, 2009). A single male-induced plant can produce around 3.5 million pollen grains, which can fertilize millions of female flower plants in a single growing season (Olejar and Park, 2022).

### 5.4 The right placement of the silver thiosulfate

The precise placement or application location of silver thiosulfate (STS) in *Cannabis* plants is crucial for effective absorption, assimilation, and sex reversal. Breeders suggest that the STS solution should be foliar sprayed onto the entire plant until runoff (Lubell and Brand, 2018; DiMatteo et al., 2020). Moreover, Flajšman et al. (2021) indicated that the efficacy of STS application is higher when the entire plant is foliar sprayed, rather than specifically applying STS to the shoot tips. These studies confirm the efficiency of foliar application of STS as the most reliable application method, offering promise to *Cannabis* cultivators for commercial application.

### 5.5 The right application frequency of silver thiosulfate

The frequency of STS application does not significantly affect the degree of masculinization in *Cannabis* plants. Studies have shown that whether STS is applied to plants in three consecutive intervals of 7 days under vegetative stage (Lubell and Brand, 2018) or with a single application followed by flower induction, all plants underwent masculinization in the same growth stage (Flajšman et al., 2021). To minimize operational costs, a single application of STS is sufficient to induce maleness in female *Cannabis* plants.

Following STS application, it is recommended to allow the plants to remain in a long photoperiod for up to 7 days before subjecting them to a short photoperiod to induce flowering. This delay is crucial to ensure proper assimilation of STS by the plant.

### 5.6 The right photoperiod for seed production

The initiation of flowering in *Cannabis* plants significantly relies on the appropriate photoperiod. Extensive research has shown that exposing *Cannabis* plants to 12-16 hours of darkness per day induces flowering (Lubell and Brand, 2018; Peterswald et al., 2023). However, a critical photoperiod of 8-12 hours of uninterrupted darkness, termed a “short photoperiod,” is typically used for successful flowering initiation, maturation, and seed production in *Cannabis* plants (Peterswald et al., 2023). A study comparing 12 and 13-hour photoperiods in two high-THC cultivars observed that exposing plants to 13 hours of light may increase inflorescence yields without decreasing cannabinoid concentrations in some *Cannabis* cultivars grown under indoor environments (Ahrens et al., 2024).

### 5.7 The right management of *Cannabis* progeny

**Figure 2:**
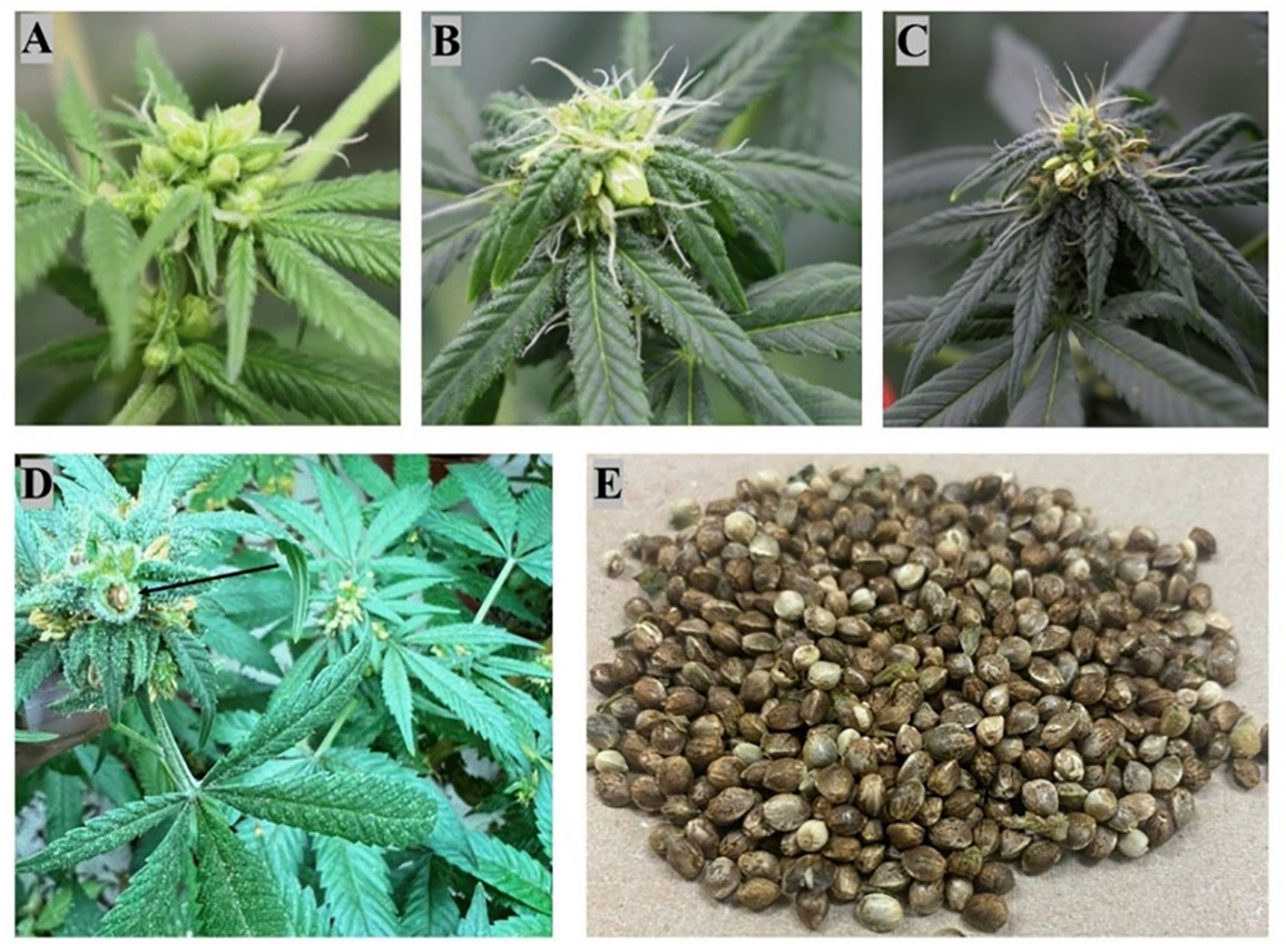
Illustration depicting the various stages of monoecious flower formation following treatment with silver thiosulfate in female Cannabis plants, including self-pollination, self-fertilization, and seed production. **(A, B)** Stigma and pollen sac development; **(C)** Pollen sacs dehisce to fertilize the stigma, causing the stigma color to change from white to brown; **(D)** Seed generation after self-pollination and fertilization; **(E)** Seed harvesting.

**Figure 3:**
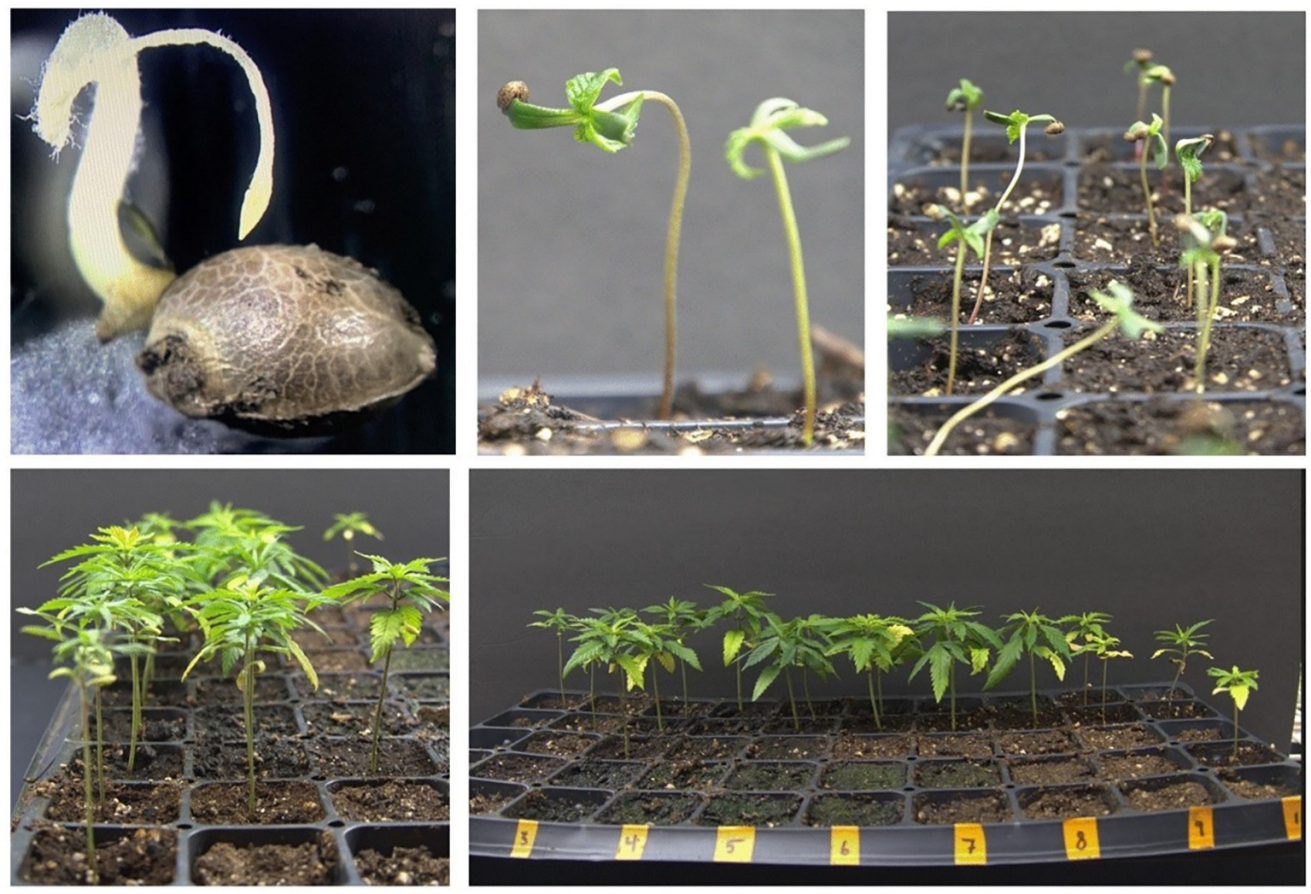
Multifaceted depiction of seed germination: exploring various developmental stages from seeds to seedling growth.

After obtaining seeds from treated female plants with silver thiosulfate as illustrated in **Figure 2**, it is imperative to conduct a thorough assessment of seed quality. This evaluation involves measuring parameters such as seed germination rate, seedling survival rate, and the expression of flowers in progeny plants (**Figure 3**). These steps are crucial to ensure the effectiveness and reliability of the applied techniques to produce feminized seeds in *Cannabis sativa* L.

In *Cannabis*, seed feminization aims to achieve female homozygosity (AA or aa) through inbreeding, potentially causing inbreeding depression, which reduces plant height, biomass, and THC yields in progeny plants due to the expression of recessive alleles (aa) (Clarke and Merlin, 2013; Kurtz et al., 2020). Evaluating potential inbreeding depression by comparing traits with those of the original parental plants is crucial for assessing its impact on progeny plants.

## 6 Conclusion

Literature research indicates that Silver Thiosulfate (STS) is the most effective compound for inducing sex reversal and feminizing seeds in high-THC *Cannabis* cultivars. Specifically, applying a single dose of 3 mM STS during the vegetative stage by foliar spraying until runoff, followed by exposure to a short photoperiod of up to 12 hours, effectively induces flowering and seed production.

Progeny plants should be evaluated for germination rates and compared to the original parent plant for growth traits, such as plant height, biomass, and Delta-9-tetrahydrocannabinol (THC) yield, to assess the negative effects of inbreeding. Following these guidelines will help growers and breeders enhance the quality and viability of feminized seeds, thereby meeting the high standards and demands of the commercial market for high-THC *Cannabis* cultivars.
